# *‘United we stand, divided we fall’:* intertwining as evidence of joint actions in pea plants

**DOI:** 10.1093/aobpla/plad088

**Published:** 2023-12-20

**Authors:** Bianca Bonato, Qiuran Wang, Silvia Guerra, Valentina Simonetti, Maria Bulgheroni, Silvia Quaggiotti, Benedetto Ruperti, Umberto Castiello

**Affiliations:** Department of General Psychology, University of Padua, Via Venezia 8, 35131, Padua, Italy; Department of General Psychology, University of Padua, Via Venezia 8, 35131, Padua, Italy; Department of General Psychology, University of Padua, Via Venezia 8, 35131, Padua, Italy; Department of General Psychology, University of Padua, Via Venezia 8, 35131, Padua, Italy; Ab.Acus s.r.l, Via Francesco Caracciolo 77, 20155, Milan, Italy; Ab.Acus s.r.l, Via Francesco Caracciolo 77, 20155, Milan, Italy; Department of Agronomy, Animals, Food, Natural Resources and Environment, University of Padua, Viale dell'Università 16, 35020, Legnaro, Italy; Department of Agronomy, Animals, Food, Natural Resources and Environment, University of Padua, Viale dell'Università 16, 35020, Legnaro, Italy; Department of General Psychology, University of Padua, Via Venezia 8, 35131, Padua, Italy

**Keywords:** Cooperation, intertwining, joint action, kinematics, pea plants, plant behavior, plant cognition, social cognition

## Abstract

In life, it is common for almost every kind of organism to interact with one another. In the human realm, such interactions are at the basis of joint actions, when two or more agents syntonize their actions to achieve a common goal. Shared intentionality is the theoretical construct referring to the suite of abilities that enable such coordinated and collaborative interactions. While shared intentionality has become an important concept in research on social cognition, there is controversy surrounding its evolutionary origins. An aspect still unexplored but promising to bring new insights into this open debate is the study of *aneural* organisms. To fill this gap, here we investigate whether climbing plants can act jointly to achieve a common goal, i.e. reaching the light. We examined *Pisum Sativum* plants growing intertwined when there is a need to climb but a potential support is not present in the environment. Three-dimensional kinematic analysis of their movement revealed a coordinated and complementary behaviour. They tend to coordinate their movement in time and space to achieve a joint climbing. By deliberately extending the context in which a joint action takes place, we pay tribute to the complex nature of this social phenomenon. The next challenge for the field of joint action is to generate a perspective that links coordination mechanisms to an evolutionary framework across taxa.

## Teaser

Through the mathematical description of movement (i.e. kinematics), we demonstrate that pea plants are able to act jointly for the achievement of a common goal, i.e. to reach the maximum exposure to light. When a support is not available, they organize a braided structure that supports their ascent towards the light. Of relevance is that their movement is highly coordinated in time and space as evidenced by correlational analyses. The movement exhibited by the two plants acting together is completely different from the one exhibited by a plant acting towards a static and inanimate support (i.e. a wooden pole). This suggests that plants are able to adapt their movement depending on the kind of element they are interacting with. Our findings provide an important contribution to the study of coordinated and collaborative interactions in climbing plants.

## Introduction

The ability to coordinate actions with those of others in time and space is essential to improve the chances of survival as individuals and as a species. Shared actions to achieve a common goal are termed *joint actions* and involve two or more agents. To act in concert during joint actions, numerous coordination problems need to be solved. For instance, initiators of the *joint action* need to make their intentions intelligible to their partners to establish a shared intentionality. Shared intentionality is an evolutionary response to the problems encountered during the coordination of a complex *joint action*, which humans (Tomasello *et al.* 2005; Enfield and Levinson 2006; Tomasello 2014) and nonhuman social animals can operationalize (Trivers 1971; Clutton-Brock 2009; Gelblum *et al.* 2015; Heesen *et al.* 2021a, b). Joint actions and shared intentionality have been broadly investigated in the animal domain (Bekoff 2001; Palagi 2006), an ideal manner to study complex cognitive abilities such as the attunement to one’s partner. Different studies examined the ability to act jointly not only in different mammalian groups (Boesch 2002; Levinson 2006; Fedurek and Dunbar 2009; Sebanz and Knoblich 2009), birds (Pozis-Francois *et al.* 2004) and dolphins (Blomqvist *et al.* 2005; Sartori *et al.* 2015) but also in insects. Ants, for example, are able to coordinate foraging using pheromones (Hölldobler and Wilson 1990; Vittori *et al.* 2006). Moreover, bacteria, are able to form rafts acting together (Kearns 2010). This shows that joint actions are possibly a product of evolution and that having central nervous systems is not a prerequisite to generating coordinated actions.

Attuning to others’ behaviours might represent an ability that has been selected and maintained through evolution among *taxa* and is necessary to organize organisms in groups to cooperate for survival. We push these concepts to the limits by expanding the study of joint actions in plants. We investigate whether plants can act jointly to achieve a common goal. Observations from numerous species of climbing plants reveal that climbing shoots often intertwine and provide mutual support within braided structures (Rowe and Speck, 2015). This is a behaviour that could be defined as a joint action.

We build on recent studies reporting that climbing plants are able to implement an anticipatory goal-directed behaviour on the basis of a potential support’ characteristics (Guerra *et al.* 2019). Once they perceived a potential support, climbing plants are able to determine its characteristics (e.g. diameter, length, distance, material) and build a coherent approach-to-grasp plan by scaling kinematical signatures accordingly (Guerra *et al.* 2019; Ceccarini *et al.* 2020a; b; see also Wang *et al.* 2023). Such studies have been conducted with individual plants acting. Bonato *et al.* (2023), instead, investigated plants implementing different kind of movement either in an individual or social context to grasp a potential support. This study confirmed that plants are able to perceive their neighbours and implement behavioural responses tuned to a diverse social attitude, either individually or socially (Bonato *et al.* 2023).

In the present study, we go a step further by asking whether climbing plants can attune their movement to that of another plant to accomplish a common goal: climbing towards the light.

This may demonstrate the plants’ ability not only to act in a social context but also to take advantage of the social affordances the environment offers.

To do this, we examined via kinematical analysis how two pea plants coordinate their actions to grow intertwined when they need to climb in the absence of a potential support. A careful kinematical examination of their movement together with correlational analyses revealed a complementary pattern of movement, with each plant of the dyad taking a specific role. We also included control conditions in which the plants move in the absence of a support or in which the support is an inanimate object.

## Results

### Experiment 1

**Figure 1.**
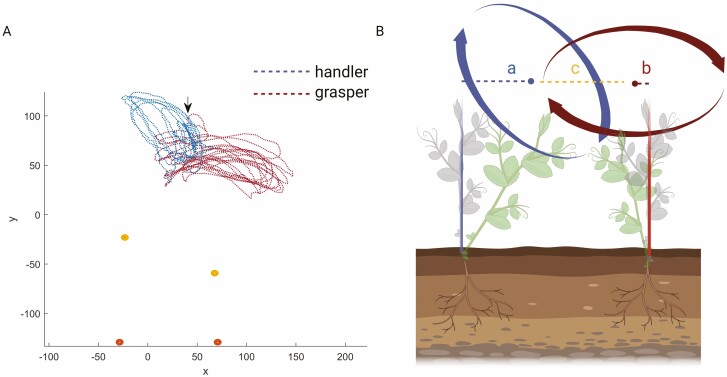
Graphical depiction of the trajectories for an exemplary couple of *handler* and *grasper* plants. Panel A represents the tendrils circumnutation trajectories. The blue line represents the circumnutation trajectory for the *handler* plant. Note that it is not perpendicular to its vertical axis but is inclined towards the other plant. The red dotted line represents the circumnutation trajectory for the *grasper* plant, ending with a grasping phase, represented by the black arrow. The orange dots represent the origin of the plants. Yellow dots represent the internode of the plants. Orange and yellow dots represent the plants’ stems. Panel B presents a graphical illustration of the ‘distance between the circumnutation center of gravity and the origin of the plant’ (a for the *handler*; b for the *grasper*) and the ‘distance from the gravity center of circumnutation to the other plant’ (c).

We collected three-dimensional (3D) kinematic data from 16 snow peas (*Pisum sativum* var. saccharatum cv Carouby de Maussane) bringing to 8 couples. When considering each couple of plants, differences in the growth pattern emerge (see Supporting Information video 1). In all cases, 1 plant, termed the *handler,* bends towards the other plant to reach it. The other plant, termed the *grasper*, deviates slightly from its central axis up to the point the *handler* is at a graspable distance (Fig. 1).

Once the *handler*’s tendrils were nearby, the *grasper* clasped them, and the two plants intertwined and climbed towards the light. In other words, the *handler* plant initiates the joint action while the *grasper* plant strategically modifies the trajectory of its tendrils to clasp those of the *handler* (Fig. 1; see Supporting Information video 1). More specifically, the *grasper* exhibits a classic circumnutation pattern perpendicular to its axis (Stolarz 2009). Instead, the *handler* exhibits circumnutations that are not perpendicular to its axis but exaggeratedly inclined towards the *grasper*. This suggests both plants exhibit a specific form of spatial navigation sub serving a common goal.

#### Kinematic results.

When looking at the kinematical patterning characterizing the handler and the grasper, we found no differences in the temporal occurrence of key kinematic landmarks (see Table 1). Rather, differences emerge at the spatial measures level. The median distance from the plant’s origin to its circumnutation centre of gravity is 53.578 mm (IQR = 64.799, range = 128.057, percentiles [19.625, 53.578, 84.4425]) for the handler and 25.675 mm (IQR = 24.788, range = 65.083, percentiles [13.225, 25.675, 38.043]) for the grasper (see Fig. 1).

**Table 1.** Bayesian Mann–Whitney *U* test for the differences between the *handler* and *grasper* plants.

Dependent measures	BF_10_	W	Rhat
Number of circumnutations	0.183	5933.500	1.001
Maximum velocity during circumnutations	0.158	5892.000	1.004
% time at which maximum velocity occurs	0.511	24.000	1.000
Mean velocity during circumnutations	0.153	5925.000	1.002
Minimum velocity during circumnutations	0.151	5826.000	1.004
Duration of the circumnutations	0.399	5034.000	1.006
Total switches	0.492	37.500	1.000
Distance from the gravity centre of the circumnutation to the origin of the plant	1669.161*	8402.000	1.065
Distance from the gravity centre of the circumnutation to the origin of the other plant	11409.445*	3179.000	1.018

*Note*. Result based on data augmentation algorithm with five chains of 1000 iterations.

**Figure 2.**
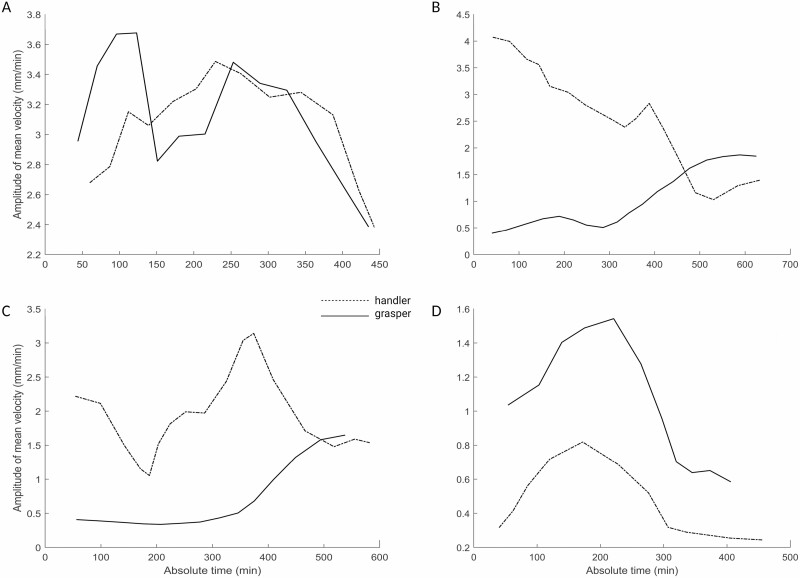
Graphical representation of the amplitude of mean velocity for representative couple of plants. Note that the velocity profile is progressively coordinated in time and becomes increasingly more similar for the two plants as the joint action progresses. The movement for the two plants ends with a progressive attunement of the velocity profiles.

The Bayesian Mann–Whitney *U* analysis revealed a Bayes factor (BF_10_) of 1669.161, suggesting there is an extreme difference between the two plants when considering the distance from the origin (BF_10_ = 1669.161, *W* = 8402.000, R-hat = 1.065). The median distance from the circumnutations centre of gravity to the origin of the other plant is 80.702 mm (IQR = 38.291, range = 102.440, percentiles [55.946, 80.702, 94.237]) for the *handler* and 93.505 mm (IQR = 22.387, range = 105.351, percentiles [86.501, 93.505, 108.888]) for the *grasper* (see Fig. 2). The Bayesian Mann–Whitney *U* analysis revealed a Bayes factor (BF_10_) of 11 409.445, suggesting there is an extreme difference between the *handler* and the *grasper* (BF_10_ = 11 409.445, *W* = 3179.000, R-hat = 1.018) for this measure. Table 4 reports all the descriptive statistics.

#### Bayesian correlations.

Kendall’ *τ* correlation determined how two values co-vary in time. This index indicates global synchrony. Here, we use this method to test a possible crosstalk between the two plants. Table 2 reports all the correlations.

**Table 2.** Bayesian Kendall’s tau correlations. BF_10_* indicates a moderate correlation; BF_10_** a strong correlation; BF_10_*** a decisive correlation.

Variable 1	Variable 2	Kendall’s tau B	BF_10_
Grasper mean velocity	Handler mean velocity	0.172	7.135*
Grasper duration circumnutation	Handler duration circumnutations	0.643	6.914*
Grasper total circumnutations	Handler total circumnutations	0.889	43.878**
Distance from the origin	Distance from the other plant	−0.296	1.561 × 10^8^***

One correlation considered is the *amplitude of the mean velocity between the two plants*. This measure indicates a kinematical harmony necessary to move in a similar and coordinated pattern (Fig. 2; Table 2). The correlation between the two plants when considering the amplitude of mean velocity is moderately positive (Table 2).

**Figure 3.**
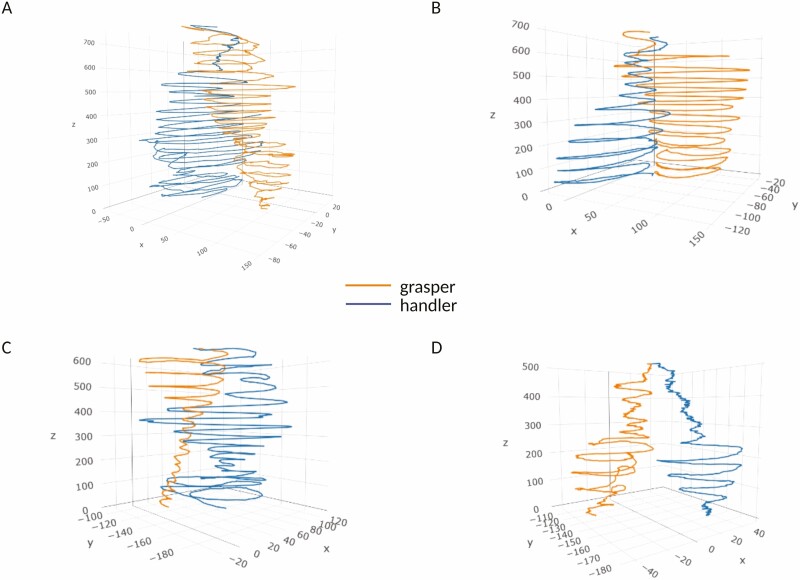
Trajectories representing the evolution in time of the distance between the handler and the grasper for representative plants. The graphs (a–d) show the gradual approach between the two plants in time, with a progressive reduction of distance between their tendrils.

Another correlation considered is between the circumnutation duration for the two plants. This measure is moderately positively correlated (Table 2). We also considered the correlation between the total *number of circumnutations* the two plants performed. The correlation was strongly positive (Table 2). For what concerns spatial coordination, the distance from the circumnutation centre of gravity to the origin of the plant and the distance from the circumnutation centre of gravity to the origin of the other plant was correlated (Fig. 1B). These two measures indicate the plants’ spatial positions and their correlation is extremely negative. This signifies a progressive estrangement of one plant from its central axis and simultaneously a progressive approach towards the central axis of the other plant (Fig. 3; Table 2).

#### Comparing the intertwining with an individual condition with no support.

We compared the behaviour of the *handler* and the *grasper* plants with the behaviour of eight plants growing in isolation in the absence of support in the environment. Results show that the plants’ behaviour for the intertwining condition differs from that the plants exhibited when acting alone (Table 3).

**Table 3.** Bayesian Mann–Whitney *U* test between the *individual* and *grasper* and *individual* and *handler* plants. Result based on data augmentation algorithm with five chains of 1000 iterations.

	Individual vs. Grasper			Individual vs. Handler		
	BF_10_	W	Rhat	BF_10_	W	Rhat
Number circumutations	5.370 × 10^+8*^	45 790.000	1.040	10 913.255*	6644.000	1.288
Maximum velocity	2313.206*	38 111.000	1.014	172.293*	10 724.000	1.068
Time % maximum velocity	2.727	55.000	1.000	4.072	6.000	1.002
Mean velocity	152 603.772*	39 024.000	1.009	2106.514*	10 247.000	1.016
Minimum velocity	12 418.432*	38 072.000	1.035	115.773*	10 668.000	1.069
Duration circumnutations	1.699 × 10^+9*^	8758.000	1.020	66 7602.473*	23 512.000	1.058
Total switches	1.202	15.000	1.001	1.165	13.500	1.000
Distance from the gravity centre of the origin of the plant	1.872	25 327.000	1.009	350.005*	19 791.000	1.027

These findings suggest that another plant in the environment is considered a potential support from the very beginning of growth. Note that the *distance from the circumnutation centre of gravity to the other plant* was not considered as a dependent measure because there is not an equivalent measure for the control condition. Table 4 reports the descriptive statistics.

**Table 4.** Descriptive statistics for the grasper, the handler and the individual plants

	Group	Median	IQR	Range	25th percentile	50th percentile	75th percentile
Number circumnutations	Grasper	7.000	7.000	18.000	4.000	7.000	11.000
	Handler	7.000	7.000	21.000	4.000	7.000	11.000
	Individual	18.000	18.000	58.000	9.000	18.000	27.000
Maximum velocity	Grasper	2.910	3.091	18.118	1.253	2.910	4.344
	Handler	2.732	2.996	13.355	1.556	2.732	4.552
	Individual	3.964	4.156	19.656	2.262	3.964	6.418
Time % maximum velocity	Grasper	67.371	43.214	76.771	45.852	67.371	89.066
	Handler	49.886	63.288	77.275	28.106	49.886	91.394
	Individual	97.727	11.130	24.806	88.375	97.727	99.505
	Grasper	1.582	1.694	4.224	0.714	1.582	2.408
Mean velocity	Handler	1.574	1.907	0.110	0.718	1.574	2.625
	Individual	2.172	2.839	6.484	1.174	2.172	4.013
	Grasper	1.582	1.694	4.224	0.714	1.582	0.890
Minimum velocity	Handler	0.473	0.732	2.493	0.183	0.473	0.915
	Individual	0.750	1.324	4.141	0.282	0.750	1.606
	Grasper	111.000	39.000	144.000	93.000	111.000	132.000
Duration circumnutations	Handler	105.000	51.000	252.000	75.000	105.000	126.000
	Individual	63.000	18.000	159.000	57.000	63.000	75.000
	Grasper	0.500	3.000	4.000	0.000	0.500	3.000
Total switches	Handler	1.500	1.250	3.000	1.000	1.500	2.250
	Individual	3.000	2.250	9.000	2.750	3.000	5.000
Distance from the gravity centre to the origin of the plant	Grasper	25.675	24.788	65.083	13.225	25.675	38.043
	Handler	53.578	64.799	128.057	19.625	53.578	84.425
	Individual	24.429	27.819	105.034	14.895	24.429	42.714
Distance from the gravity centre to the origin of the other plant	Grasper	93.505	22.387	105.351	86.501	93.505	108.888
	Handler	80.702	38.291	102.440	55.946	80.702	94.237
	Individual	–	–	–	–	–	–

### Experiment 2

To disentangle the role of social affordances, we conducted a second experiment comparing the behaviour of plants moving towards either a conspecific (plant–plant condition) or an inanimate object (plant–object condition).

#### Kinematic results.

**Figure 4.**
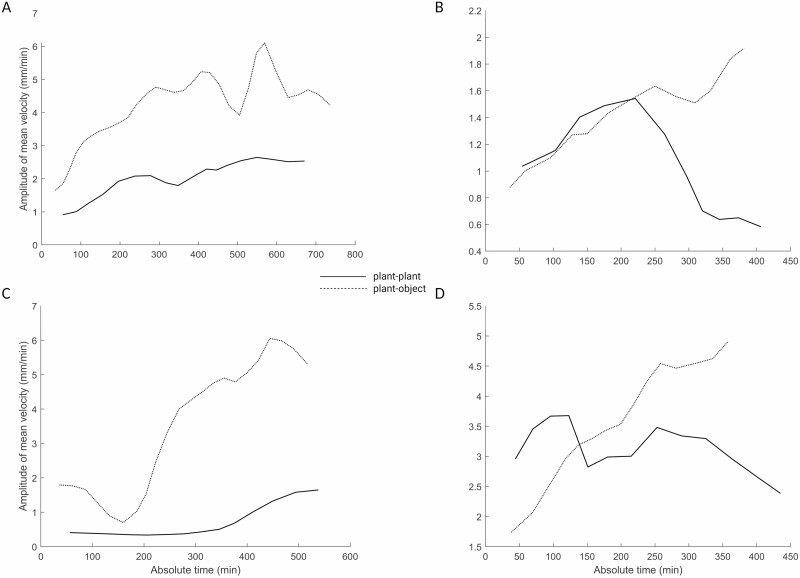
Graphical representation of the amplitude of mean velocity for representative plants in the plant–plant and plant–object condition. Note that the velocity profile is completely different for the plants acting in the two conditions. The plants’ movement towards an object presents a higher velocity, with higher acceleration peaks until the end of the movement time.

The kinematic pattern between the plant–plant and plant–object conditions revealed significative differences in key kinematical signatures (see Tables 5 and 6). The significative difference between the two conditions is evident when we consider the peak velocity. The velocity profile is characterized by many peaks of acceleration and deceleration (see Table 5; Fig. 4). The number of circumnutations required to accomplish the grasping phase is lower for the plant-plant than the plant–object condition (see Table 5). In spatial terms, the plants moving towards a conspecific (i.e. plant–plant condition) showed a lower inclination from its central axis towards the other plant, and at the same time a similar inclination towards the stimulus with respect to the plant–object condition (see Table 5). This could be explained by the progressive approaching by the other plant moving in the dyad for the plant-plant condition.

**Table 5.** Descriptive statistics for the dependent measures considered between the two conditions.

		Median	IQR	Range	25th percentile	50th percentile	75th percentile
Total circumnutation	plant_plant	7.00	7.00	18.00	4.00	7.00	11.00
	plant_object	14.00	20.00	57.00	7.00	14.00	27.00
Max_speed_circumnutations	plant_plant	2.91	3.09	18.11	1.25	2.91	4.34
	plant_object	5.60	3.39	12.08	4.05	5.60	7.44
Time % at which max_speed	plant_plant	60.82	45.52	95.78	30.49	60.82	76.02
	plant_object	69.62	45.33	99.93	31.27	69.52	76.69
Mean_speed_circumnutations	plant_plant	1.58	1.69	4.27	0.71	1.58	2.40
	plant_object	3.20	2.50	6.24	2.09	3.20	4.59
Min_speed_circumnutations	plant_plant	0.35	0.74	1.99	0.14	0.35	0.89
	plant_object	1.20	1.72	4.51	0.53	1.20	2.26
Duration_circumnutation	plant_plant	111.00	39.00	144.00	93.00	111.00	132.00
	plant_object	69.00	21.00	90.00	60.00	69.00	81.00
Total switches	plant_plant	0.50	3.00	4.00	0.00	0.50	3.00
	plant_object	1.500	3.000	4.00	0.00	1.50	3.00
Distance_from_origin	plant_plant	25.67	24.78	65.08	13.25	25.67	38.04
	plant_object	45.53	48.17	105.69	28.72	45.53	76.90
Distance_from_stimulus	plant_plant	93.50	22.38	105.35	86.50	93.50	108.88
	plant_object	92.81	28.09	153.65	79.41	92.81	107.50

**Table 6.** Bayesian Mann–Whitney *U* test for the *plant–plant* and *plant–object* condition. Result based on data augmentation algorithm with five chains of 1000 iterations.

	BF_10_	*W*	Rhat
Total circumnutations	32 272.846	5768.500	1.039
Max_speed_circumnutations	6.476 × 10^+6^	4012.000	1.056
Time % at which max_speed	0.914	1941.500	1.002
Mean_speed_circumnutations	255 862.518	3779.000	1.239
Min_speed_circumnutations	3.295 × 10^+9^	4604.000	1.157
Duration_circumnutation	1.722 × 10^+7^	18 996.000	1.111
Total switches	0.455	29.000	1.000
Distance_from_origin	57 364.337	4696.000	1.093
Distance_from_stimulus	0.358	11 669.000	1.057

Unlike the results from Experiment 1, the results from Experiment 2 show that the movement towards a conspecific presents a more careful honing phase and a lower velocity, possibly allowing for better synchronization with the other plant’s movement (see Fig. 4). These results disentangle the ‘social affordance’ issue, supporting the idea that what we unveiled in Experiment 1 a cooperative attitude mediated by a shared ‘intentionality’ to achieve a goal.

#### General discussion.

In the present study, we investigated for the first time whether plants can act jointly and whether some forms of shared intentionality are at the basis of their ‘*intertwining*’ behaviour. Results revealed specific motor patterns for the two plants in the dyad. Evidence from correlational analyses demonstrate that *aneural* organisms can act jointly and not simply together.

Looking at the results, we can immediately appreciate the two plants’ non-casual behaviour during the intertwining interaction (see Supporting Information video 1). This supports the idea that pea plants move in a flexible and anticipatory manner (Guerra *et al.* 2019; Ceccarini *et al.* 2020a; b; Bonato *et al.* 2023; Wang *et al.* 2023). We explain these effects in terms of affordances. That is the quality or property of an object that defines its possible uses or clarifies how it can or should be used (Gibson 1977). The theory of affordances proposes that there is enough information in the environment to make sense of the world in a direct way. In this perspective, the environment surrounding an organism is perceived for action, and an organism will perceive something for its affordances rather than its qualities. Affordances are also an essential part of socialization. Social affordances, a subcategory of affordances, provide the opportunities in the environment to promote social relationships and interactions (Becchio *et al.* 2008; Ferri *et al.* 2011). In the case of our plants, the intertwining phenomena represent a perfect exemplification of the social affordance concept. The pea plants perceived each other as a potential support and then acted in concert. This is witnessed by how the two plants coordinate their action in time to meet at a precise point in space to reach kinematical consonance and by how these motor patterns are totally different with respect to the one executed by a plant acting towards an inanimate object, as demonstrated in Experiment 2 (see Supporting Information video 3).

Kinematical consonance serves as an index to demonstrate that plants act jointly and not simply together. To elaborate, in humans’ movement, attunement may make the interacting partners more similar and thus more predictable to one another (Keller *et al.* 2007; Vesper *et al.* 2010). Here, we show that this can also happen in plants. Our findings suggest that the pattern of movement is the very same for the two plants. To reach such a level of coordination, agents need to solve numerous coordination problems via ‘coordination smoothers’. For an agent, one way to facilitate coordination is to modify its behaviour to make it easier for others to predict upcoming actions, for example, by exaggerating the movements or by reducing the actions’ variability (Vesper *et al.* 2010). Another example of coordination smoother is the assignment of tasks between partners (Vesper *et al.* 2013; Skewes *et al.* 2015). From the present results, it is evident that the two plants in the dyad manifest specific but complementary behavioural patterns. The *handler* plant bends exaggeratedly towards the *grasper* to facilitate intertwining and then they travel together towards the light. Therefore, it seems that the initiator of the joint action is the *handler.* It signals the other plant on the potential common goal, and it coordinates the action. The possibility that each plant plays a specific role suggests we are not in the presence of an ‘imitative’ behaviour, but a complementary behaviour driven by a shared goal, requiring cooperation and some forms of shared intentionality. These are two plants taking two roles, it is not simply an ‘action performed together’. The exaggerated behaviour of the *handler* towards the neighbour could be explained as a coordination smoother to render intentions intelligible. Further, this pattern differs hugely from that exhibited by the control group of plants moving without supports (see Supporting Information video 2) means this is a type of behaviour that is enormously exaggerated regarding the plants’ usual behaviour.

Another interesting result concerns the progressive approach between the two plants (Fig. 3). The correlational analysis allows appreciating a non-casual correlation between the progressive distance between the origin of the plant and the gravity centre of the circumnutations and the distance between the gravity centre of the circumnutations to the origin of the other plant. This signifies they do not approach each other casually. When plants circumnutate, they perform an elliptical rotatory movement that allows for exploring each sector of the proximal environment. For the intertwining condition of our study, the plants’ progressive distance from their original axes is functional to reach the point in space where the other plant is placed. This negative correlation occurring between the above-mentioned dependent measures witnesses this.

Altogether, these strategies seemed to be aimed at saving energy. The basis of the processes described here is high adenosine triphosphate (ATP) consumption (Putz and Holbrook 1992). Fewer circumnutations, fewer switches in direction, and a lower velocity may allow the two plants to preserve energy to reduce the risks of errors and attach firmly to each other. Remember that plants in the control group (those acting in isolation with no supports) show more circumnutations and switches in direction than the intertwining plants. This strategy is further supported by the ‘velocity’ results, which show that the *intertwining* plants exhibit a longer deceleration phase than the plants acting in isolation do. Further, remember the plants in Experiment 2 (those acting with an inanimate object) show more circumnutations and switches in direction than the plant moving towards another plant in the plant–plant condition, such as the control condition of an individual plant without support with respect to the intertwining condition of Experiment 1. This suggests that social actions require a more careful movement patterning for the sake of the necessary monitoring when acting jointly (Vesper *et al.* 2017). This signifies that acting together or acting in isolation reflects on kinematic patterning. Ascribing a specific *social affordance* to the other plant, and not simply perceiving it as a neighbour or a passive support might be taken as evidence of implementing the shared intention to intertwine and grow together towards the light.

A final aspect of the present findings relates to movement duration, which is longer for the intertwining than for the control group in Experiment 1 and the plant–object condition in Experiment 2. For the intertwining plants, the extra time needed may allow for better control of the tendrils’ trajectories and a more accurate selection of contact points to twine firmly around the neighbour. This interpretation is consistent with the most prominent theory of speed accuracy-trade off (Meyer *et al.* 1988) recently confirmed in plants (Ceccarini *et al.* 2020a).

To conclude, we provide here the first empirical account of the intertwining behaviour. We situate our findings within available theories explaining joint and complementary actions in animal species. This is not to make plants resemble animals but to say that in aneural organisms, movements with a shared intentionality drive are possible. This calls for a reformulation of traditional definitions of shared intentionality based on concepts belonging to sometimes arbitrary and limited conceptions. Everything now must find empirical confirmation not only at the behavioural but also at the physiological level and needs to be done through species-specific tests under the banner of a pluralistic interdisciplinary approach, open to future breakthroughs and additions.

#### Limitations and future directions.

In this study, we observed two plants acting together to accomplish the same goal.

A limitation of the study is that we did not consider an intertwining experiment with two plants potted in two distinct pots to avoid communication between roots. This condition would provide hints regarding the possibility of whether and how is possible for plants to act jointly when the communication between roots is not allowed.

Researchers should consider a deeper investigation of the chemical compounds the plants release during these social interactions to merge kinematic and physiological analyses, thereby embracing a multidisciplinary approach to the study of this complex and fascinating behaviour. Further, it would be useful to integrate these results with ecological observations in open fields to gain a better understanding of the delicate equilibrium of ecosystems.

## Materials and Methods

### Experiment 1

#### Subjects.

Twenty-four snow peas (*Pisum sativum* var. saccharatum cv Carouby de Maussane) were chosen as the study plants. Pea seeds were germinated, potted, and kept at the conditions outlined below. We determined our sample size based on our previous studies (Guerra *et al.* 2019; Ceccarini *et al.* 2020a; b; Bonato *et al.*, 2023; Wang *et al.*, 2023).

#### Experimental conditions.

**Figure 5.**
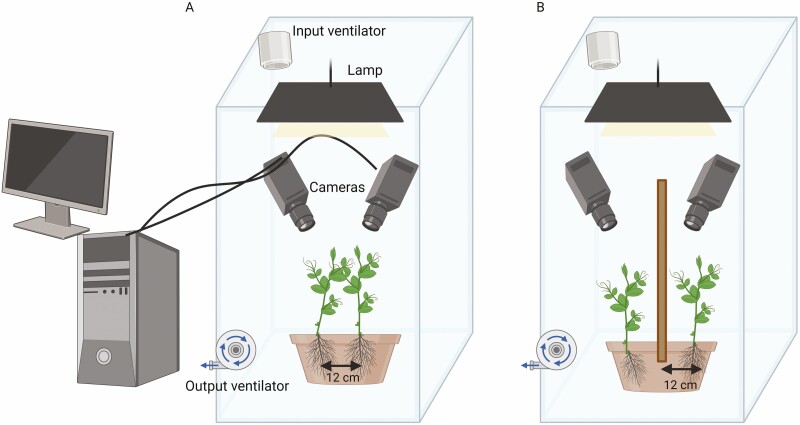
Graphical representation of the experimental setups. Panel A illustrates the experimental setup of Experiment 1 for the intertwining condition. Panel B illustrates the experimental setup for the plant–object condition in Experiment 2.

A condition in which two pea plants grew within the same pot without the presence of potential support in the environment was considered (see Fig. 5A). In such circumstances, the plants were somewhat constrained to intertwine to climb towards the light. A control condition in which a single pea plant grew in a pot without the presence of potential support in the environment was also considered. Experiment 2 reported also the plant–object condition in which a pea plant grew in the pot with the presence of an inanimate object (i.e.i.e. wooden pole; see Fig. 5B). Treatments were replicated eight times by randomly assigning the two plants’ locations within the pot.

Germination and growth conditions. The seeds were made to germinate in absorbent paper for 6 days and then the healthy and same-rate height plants were potted. The pot used was 30 cm in diameter and 14 cm in height. The pots were filled with silica sand (type 16SS, dimension 0.8/1.2 mm, weight 1.4). At the beginning of each treatment, the pots for the individual condition were watered and fertilized using a half-strength solution culture (Murashige and Skoog Basal Salt Micronutrient Solution; 10x, liquid, plant cell culture tested; SIGMA Life Science). The soil volume and the solution culture allowed for adequate soil and fertilizing conditions for the plants in the control condition and they have been adjusted to allow the same quantity of soil and fertilizer for the two plants in the intertwining condition, with a suitable bigger pot. The plants were watered 3 times a week. Each pot was enclosed in a growth chamber (Cultibox SG combi 80 × 80 × 160 cm) so the plants could grow in controlled environmental conditions. The chamber air temperature was set at 26 °C and remained constant between 24 °C and 26 °C during the day–night cycle; the extractor fan was equipped with a thermo regulator (TT125; 125 mm-diameter; max 280 MC/H vents) and there was an input-ventilation fan (Blauberg Tubo 100–102 m^3^/h). The 2-fan combination allowed for a steady air flow rate into the growth chamber with a mean air residence time of 60 s. The fan was placed so that air movement did not affect the plants’ movements. Plants were grown with an 11.25-hr photoperiod (5.45 am to 5 pm) under a cool white LED lamp (V-TAC innovative LED lighting, VT-911-100W, Des Moines, IA, USA or 100W Samsung UFO 145lm/W—LIFUD) that was positioned 50 cm above each seedling. Photosynthetic Photon Flux Density at 50 cm under the lamp in correspondence with the seedling was 350 μmol_ph_/m^2^s (quantum sensor LI-190R, Lincoln, Nebraska USA). Reflective Mylar^®^ film of chamber walls allowed for better uniformity in light distribution (Figure 5).

#### Video recording and data analysis.

For each growth chamber, a pair of RGB-infrared cameras (i.e. IP 2.1 Mpx outdoor varifocal IR 1080P) were placed 110 cm above the ground, spaced at 45 cm to record stereo images of the plant. The cameras were connected via Ethernet cables to a 10-port wireless router (i.e. D-link Dsr-250n) connected via Wi-Fi to a PC and the frame acquisition and saving process were controlled by CamRecorder software (Ab.Acus s.r.l., Milan, Italy). To maximize the contrast between the peas’ anatomical landmark (e.g. the tendril) and the background, black felt velvet was fixed on some sectors of the boxes’ walls. The intrinsic, extrinsic, and lens distortion parameters of each camera were estimated using a Matlab Camera Calibrator App. Depth extraction from the single images was conducted by taking 20 pictures of a chessboard (squares’ side 18 mm, 10 columns, 7 rows) from multiple angles and distances in natural non-direct light conditions. For stereo calibration, the same chessboard used for the single-camera calibration process was placed in the middle of the growth chamber. The two cameras took the photos to extract the stereo calibration parameters. In accordance with the experimental protocol, the camera synchronously acquired a frame every 3 min (frequency 0.0056 Hz). The tendrils developing from the considered node were studied. In those cases, in which the plant grasped the stimulus, the coiled leaf was analysed. The initial frame was defined as the frame in which the considered leaf’s tendrils were visible from the apex. The end of the plant movement was defined as the frame in which the leaf’s tendrils started to coil around the other plant’s tendrils. An ad hoc software (SPROUT, Simonetti *et al.* 2021) developed in Matlab and Python was used to identify anatomical points to be investigated via markers and to track their position frame by frame on the images the two cameras acquired to reconstruct the 3D trajectory of each marker. The markers on the anatomical landmark of interest, namely the tip of the tendril, were inserted post hoc (Fig. 1). The tracking procedures were at first performed automatically throughout the time course of the movement sequence using the Kanade-Lucas-Tomasi algorithm on the frames each camera acquired after distortion removal. The tracking was manually verified by the experimenter, who checked the position of the markers frame by frame. The 3-D trajectory of each tracked marker was computed by triangulating the 2-D trajectories obtained from the 2 cameras (Fig. 1).

Dependent measures. The dependent variables specifically tailored to test our experimental hypothesis on the basis of previous kinematical studies on approach-to-grasp in pea plants (e.g. Simonetti *et al.* 2021) were: (i) the spatial trajectories designed by the tip of the tendril (Fig. 1), (ii) the tendril’s total number of circumnutations, (iii) the maximum velocity of the tendril during circumnutations, (iv) time in percentage (%) at which the peak of maximum velocity occurs, (v) the mean velocity of the tendril during circumnutations, (vi) the minimum velocity of the tendrils during circumnutations, (vii) the duration of the circumnutations, (viii) the total number of the switch of the circumnutations in a clockwise or counterclockwise direction, (ix) the distance between the gravity centre of the *circumnutation* and the origin of the plant (Fig. 2), and (x) the distance between the gravity centre of the circumnutation and the origin of the other plant (Fig. 2).

Statistical analysis. The descriptive statistics including median, interquartile range (IQR), range, and percentiles (25th, 50th, and 75th) have been calculated. Statistical analyses were conducted using the Bayesian approach. The objective of Bayesian estimation is to allocate credibility to a distribution of alternative parameter values (posterior distribution) that is consistent with the observed data, by generating a large number of samples using the Markov chain Monte Carlo approach (MCMC). In this study, we adopt the two-sided Bayesian Mann–Whitney *U* test because the dependent variables are not normally distributed. Mann–Whitney *U* test is a non-parametric test that does not require the assumption of normality. The analysis was performed using JASP (JASP Team 2023) nested within the environment R (R Development Core Team 2010; see used packages: https://jasp-stats.org/r-package-list/). We choose the default prior defined by a Cauchy distribution centred on a zero-effect size (*δ*) and a scale of 0.707 (van Doorn *et al.* 2021). Data augmentation is generated with five chains of 1000 iterations that allow for simpler and more feasible simulation from a posterior distribution. In the analysis, *W* is calculated in the Mann–Whitney *U* test as the smaller of the rank total between the two conditions. Bayes factor (BF) is obtained to quantify the relative predictive performance of the two hypotheses (van Doorn *et al.* 2021). In our study, BF quantifies evidence for the presence or absence of the difference between the two conditions examined in Experiments 1 and 2. The null hypothesis here is that there is no difference in kinematics between the analysed conditions. The alternative hypothesis is that there is a difference. The BF10 value is the likelihood of data given the alternative hypothesis (H1) divided by the likelihood of data given the null hypothesis (H0). The results are reported based on Jeffery’s scheme that proposes a series of labels for which BF values can be considered either ‘no evidence’, ‘anecdotal (1–3)’, ‘moderate (3–10)’, ‘strong (10–30)’, ‘very strong (30–100)’, or ‘decisive (>100)’ relative evidence for alternative hypothesis (Jeffrey 1988). R-hat is also reported to check the degree of convergence of MCMC algorithms based on outcomes stability. The closer the value of R-hat is to 1, the better convergence to the underlying distribution. Credible intervals are set as 95%, which is simply the central portion of the posterior distribution that contains 95% of the values. We also performed Bayesian correlation for non-parametric data using Kendall’s *τ* correlation. The analyses were performed using JASP (JASP Team 2023). Kendall’s *τ* is one of the most widely used nonparametric tests of dependence between two variables (Kendall 1938). Moreover, Kendall’s *τ* expresses dependence regarding monotonicity instead of linearity and is therefore invariant under rank-preserving transformations of the measurement scale (Kruskal 1958; Wasserman 2006).

### Experiment 2

Material and methods are identical to Experiment 1, except for the following details.

#### Subjects.

Sixteen snow peas (*Pisum sativum* var. saccharatum cv Carouby de Maussane) were chosen as the study plants.

#### Experimental conditions.

Plant–plant condition. A condition in which two pea plants grew in the same pot without the presence of potential support in the environment (Fig. 5A). In such circumstances, the plants were somewhat constrained to intertwine to climb towards the light.

Plant–object condition. A condition in which a single pea plant grew in a pot in the presence of an inanimate and fixed object in the environment.

Treatments were replicated eight times by randomly assigning the two plants’ locations within the pot.

## Supporting Information

The following additional information is available in the online version of this article –

Video 1: Video concerning the intertwining behaviour exhibited by the plants.

Video 2: Video concerning the circumnutative behaviour of a plant searching for a potential support in the environment.

Video 3: Video concerning the circumnutative behaviour of a plant reaching and grasping an inanimate object (i.e.i.e. a wooden pole) as the plant-object condition reported in Experiment 2.

plad088_suppl_Supplementary_Video_S1

plad088_suppl_Supplementary_Video_S3

plad088_suppl_Supplementary_Video_S2

## Data Availability

The data underlying this article are available in www.zenodo.org repository (https://zenodo.org/records/10400719).
